# On the nature and structure of epistemic injustice in the neglected tropical disease knowledge ecosystem

**DOI:** 10.1371/journal.pntd.0012781

**Published:** 2024-12-31

**Authors:** Soumyadeep Bhaumik

**Affiliations:** 1 Meta-Research and Evidence Synthesis Unit, Health Systems Science, The George Institute for Global Health, Sydney, Australia; 2 The George Institute for Global Health, Delhi, India; 3 Faculty of Medicine and Health, University of New South Wales, Sydney, Australia; 4 Department of Public Health, Walter Sisulu University, Mthatha, South Africa; Federal University of Ceará, Fortaleza, BRAZIL

## Introduction

The phrase “knowledge is power” is widely recognised across cultures and throughout history. However, power precedes the creation of knowledge. For example, an American doctor, Samuel A. Cartwright, when entrusted with the power by the Medical Association of Louisiana, to understand specific health issues in Black people, created “knowledge” on two new diseases: *drapetomania* (a condition supposedly causing enslaved people to run away from their master) and *dysaesthesia aethiopica* (a disease that made enslaved people lazy and caused skin lesions) [1]. These findings look ridiculous now due to its dehumanising nature. But in those times, it was published in a scholarly medical journal. Objectivity, which many scientists take pride in, is essentially the inability of scientists to recognise how social power creates, drives, and sustains academia. Scientific accounts that tie objectivity to generalisability lead to delegitimisation of local knowledge [2]. They create a dichotomy wherein knowledge developed through “standard methods,” which only those with power can adhere to, is held in contrast against local knowledge. It is this dichotomy which perpetuates marginalisation of people through the power of knowledge.

The relationship between power and knowledge has been long studied, but it was Miranda Fricker who first put a name to specific form of injustice that is committed in a person’s capacity as a knower (i.e., involved in the production, use, or circulation of knowledge)—epistemic injustice [3], leading to its wider recognition. Epistemic injustice is categorized into two types:

1. Testimonial injustice—“Wherein a speaker receives an unfair deficit of credibility from a hearer owing to prejudice on the hearer’s part [3].” A non-academic example of this is the police asking for a higher burden of proof when allegations are being made by a minority group against a person from the majority group, compared to when a person of a majority group makes allegations against a person from a minority group.
2. Hermeneutical injustice—“Wherein someone has a significant area of their social experience obscured from understanding owing to prejudicial flaws in shared resources for social interpretation [3].” A non-academic example of this is a person not recognising violence in a coercive relationship due to social and cultural norms and prejudices on how personal relationships are defined in the community.

The fact that tropical medicine as a field developed with the intent to uphold and protect the financial value of colonial empires is well recognised [4,5]. As such, tropical medicine has its origin in epistemic wrongs. But with the end of the colonial era in the world, can we say that epistemic wrongs and consequential injustices are a thing of the past?

In this piece, I lay forth how epistemic injustice is still prevalent in the neglected tropical disease (NTD) research space, with the aim of provoking discussions, debates, and reforms. I outline how and why disarming epistemic injustice is not only a moral and ethical cause but also critical for better science, and for ensuring the health and well-being of individuals, communities, and nations.

## Epistemic injustice in neglected tropical disease space

The NTD research ecosystem continues to mirror a feudal structure, much like the ecosystem in global health [6]. Key global health funders continue to fund research led by tropical medicine institutes in the UK, Europe, and North America. Organisations in these non-endemic nations, where NTDs are mostly non-existent, were recipients of 75% (47,997 of a total of 63,537 million USD) of direct research funding on NTDs and 70% (40,940 of a total of 58,223 million USD) of indirect research funding on NTDs between 2007 and 2022 [7]. These funds enable the institutes to continue to build on and modernise their research infrastructure, thus keeping the colonial essence of tropical medicine alive. It is not uncommon for tropical disease research institutes to come to low-income countries to “collect samples” and data [8], using local experts as data collectors and heading back to their institutes to write research papers, build careers, and churn out more grants—thus permeating the vicious cycle.

To counter criticism of “helicopter research” [9] being promoted, global funders push impressionistic claims (not supported by data): arguments around corruption, accountability, and capacity deficits in endemic countries, which reflect hermeneutical and testimonial deficits. If the claim of these deficits is indeed true—is it not what they are supposed to be addressing! At the end of the day, the work must be and is in fact implemented by endemic country institutions in low- and middle-income countries (LMICs). Adding a layer of non-endemic country institutional bureaucracy on top of it does nothing but obfuscate administrative and financial monitoring. The systems-level denial of opportunities to lead NTD projects translates to a substantial career impact for those in endemic LMICs. It leads to the sustenance of the lack of credibility or “merit” to lead and develop agendas on tropical disease in the global space. Experiential knowledge is deemed to be less credible. On the other hand, empirical case studies from ethnobiology and traditional fishing communities examining the intersection of objectivity and local knowledge illustrate that community knowledge systems rely on causal explanations and feedback loops, unearthing varying levels of complexity [2,10]. These are often aspects that “objective” research cannot unearth because of its inherent boundaries of scope. And yet, they are not considered knowledge—an example of testimonial injustice.

Research published by scientists from LMICs and research published in local journals are deemed to be less credible. A policy analysis of how snakebite was prioritised in the WHO found that legitimate concerns from African Society of Venimology (ASV) regarding the sub-optimal engagement of African experts were dismissed as attempts to create controversy [11]. This also had significant effects—issues around the intellectual property of snake anti-venoms, which is of high concern in Africa, were never addressed. In a just ecosystem, experts from Africa and South Asia (where the highest burden of snakebite is) would not be invited, but would lead  global initiatives on snakebite. Unfortunately, this is not true for snakebite alone. This is true for the entire NTD space. Many experts in non-endemic nations do not realise their significance and contribution in the space. They are caught in the web of trying to attain metrics around publications, grants, and “impact” defined by the same “global” systems that deny their attendance. Thus continues the cycle of hermeneutical injustice.

If we dissect the concept and definition of NTD with an epistemic knife, we see it has two fundamental elements: the notion of neglect and the interplay between the disease and the cycles of poverty and development [12]. Both these elements have been crucial to garner attention and spruce this collective group of diseases into the global agenda [11,12]. We know now that poverty and development are related to every disease (not just NTDs), and it is not clear what level of neglect is appropriate to qualify as an NTD. Such a definition incentivises actors to sustain a certain level of neglect—only to keep their own interests alive. The real-world implication of such a conceptualisation of NTDs has also meant that within NTDs, only those which can be eliminated or eradicated by “magic-bullets” (preventive chemotherapy) have been prioritised and funded by global funders [9,10]. Some like snakebite, had to project anti-venoms, a curative intervention, as the magic bullet to get space in the NTD list [11]. But more recently, Noma found space without the necessity to identify a magic bullet- a welcome change. However, many other diseases continue to struggle to find space in the NTD community. The inconsistencies and lack of clarity in how the WHO operationalizes the clause requiring a disease to be “immediately amenable to broad control, elimination, or eradication” to be defined as an NTD need to be re-evaluated [11]. The normative function of WHO [13] implies they are responsible for ensuring epistemic justice in the global knowledge ecosystem.

Paradoxically, despite the framing of NTDs being centred around poverty and development, the majority of leading actors in the space as well as key interventions, strategies, and programs around NTDs, do not focus on poverty alleviation, housing, or health systems strengthening. The absence of actors and activists from endemic nations in framing priorities for NTDs has contributed to the paradox being sustained. Ameliorating NTDs demand we do good science that addresses issues in the long-term instead of investing in quick-fix “magic bullets” that are not sustainable.

## The way forward

The just NTD knowledge ecosystem, which we strive for, is not just a dream. It is a necessity to address the burden of NTDs. There have been calls for decolonisation in the global health space, which suffers from the same malice. Suggested pathways for ameliorating epistemic injustice include but are not limited to, diversifying leadership, adopting deliberative epistemic repair (giving higher credibility to marginalised knowers and avoiding practices that undermine their interpretive roles), restructuring capital flows in knowledge ecosystems, enhancing transparency in processes, co-design and co-production of research, and implementing anti-oppressive teaching [6,9,14–17]. As such, adding to this growing list of recommendations for global funders, governments, multilaterals, research institutions, journal publishers, researchers, and editors in the NTD space is an enticing prospect. However, one might argue that most of these strategies are targeted towards enhancing distributive, procedural, retributive, and restorative justice, where addressing epistemic injustice might be a side product.

To find our collective path, we need a thorough understanding of how different knowledge systems operate. We need to understand how different communities receive, process, and use knowledge from other communities. Additionally reforming complex systems in which you and I are part of, and benefit from, is long and arduous. What is critical is a better understanding of the nature and structure of injustice in the NTD knowledge ecosystem. Meta-research unearthing the diversity of epistemology in the NTD, public health, and medicine knowledge ecosystem and use of pro-justice approaches to create new knowledge is crucial. By examining how knowledge is prioritised, produced, validated, and disseminated, we can better understand the power dynamics and biases that influence scientific research, practice, and policies. The success of the generational project to disarm injustice in the knowledge ecosystem largely depends on how effectively key actors in the NTD space can support a critical mass of thinkers, meta-researchers, and health policy analysts. These individuals and organisations can drive a movement where diverse knowledge systems co-exist and interact to create new knowledge. The knowledge ecosystem would mirror threads of distinct colours being weaved together to form a beautiful design; each strand of knowledge being intertwined in a manner that they contribute to the richness and complexity of the whole. This is akin to how a person becomes well-rounded when raised by a community of Aunties, even though the mother remains the primary anchor; our understanding deepens as we continue weaving within and across diverse knowledge systems.

Post Script: In Indigenous communities of Australia, the term “Aunty” is used affectionately for older women who play a significant role in the community by contributing to the holistic development of young people.
